# *In vitro* Bioaccessibility and Intestinal Absorption of Selected Bioactive Compounds in *Terminalia ferdinandiana*

**DOI:** 10.3389/fnut.2021.818195

**Published:** 2022-01-27

**Authors:** Saleha Akter, Rama Addepalli, Michael Netzel, Ujang Tinggi, Mary Fletcher, Yasmina Sultanbawa, Simone Osborne

**Affiliations:** ^1^ARC Industrial Transformation Training Centre for Uniquely Australian Foods, Center for Nutrition and Food Sciences, Queensland Alliance for Agriculture and Food Innovation (QAAFI), The University of Queensland, Indooroopilly, QLD, Australia; ^2^Commonwealth Scientific and Industrial Research Organization, Agriculture and Food, St Lucia, QLD, Australia; ^3^Queensland Alliance for Agriculture and Food Innovation (QAAFI), The University of Queensland, Health and Food Sciences Precinct, Coopers Plains, QLD, Australia; ^4^Queensland Health Forensic and Scientific Services, Coopers Plains, QLD, Australia

**Keywords:** *Terminalia ferdinandiana*, Kakadu plum, ascorbic acid, calcium, ellagic acid, oxalate, bioaccessibility

## Abstract

*Terminalia ferdinandiana* (or Kakadu plum), a native Australian fruit with potential health benefits, contains bioactive compounds such as ellagic acid (EA), ascorbic acid (AA) and calcium, and antinutrients such as oxalic acid (OA). However, few is known about the biological fate of these compounds following ingestion; therefore, the aim of this study was to evaluate *in vitro* bioaccessibility and intestinal absorption of *T. ferdinandiana* compounds using the INFOGEST static digestion model and Caco-2-HT29-MTX-E12 intestinal absorption model. No significant changes (*p* > 0.05) were observed in total AA content throughout *in vitro* digestion, whereas bioaccessibility of EA, OA, and calcium increased significantly from 33, 72, and 67% in the gastric phase to 48, 98, and 90% in the intestinal phase, respectively. The intestinal absorption study revealed variable rates of movement across the cell barrier. Findings reveal novel and important insights for the prediction of *in vivo* bioavailability of selected *T. ferdinandiana* compounds.

## Introduction

Fruits are good sources of a range of biologically active (bioactive) compounds including fiber, phenolic compounds, vitamins, and minerals. Fruit phenolic compounds and vitamins possess strong antioxidant capacities (1) and may reduce the risk of some types of cancer, and also cardiovascular, neurodegenerative, and inflammatory diseases (2). Minerals found in fruit, such as calcium, help facilitate muscle contractions and blood clotting and contribute to strong bones and teeth. However, the bioactivity of polyphenols, vitamins, and minerals is limited by bioaccessibility that determines what proportion of these compounds are released from digested food and made available for intestinal absorption and circulation to different tissues (3). Interactions among food matrices and polyphenols, vitamins, and minerals during digestion have been extensively studied in the past decade (4) revealing that food matrix structure and composition impact bioaccessibility, digestibility, and antioxidant capacity of those compounds (3).

From a nutritional perspective, bioaccessibility is defined as the maximum fraction of a food compound that is released from the food matrix in the gastrointestinal tract that becomes available for intestinal absorption (5–7). Bioavailability is defined as the fraction of an ingested compound available for utilization in normal physiological functions (5, 6) and is a critical feature in assessing the role of dietary components in human health (8). Bioactivity refers to systemic circulation of the compound, delivery to a target tissue, and interaction with biomolecules in the tissue followed by a cascade of physiological effects (5). Thus, bioaccessibility is the first step toward achieving bioactivity from a specific compound; therefore, it is important to ascertain the nutritional quality of a nutrient or bioactive compound, not only in terms of quantities required to achieve nutritional requirements, but also to refine the development of functional foods (5).

*In vitro* assays predict intestinal absorption of food components and involve *in vitro* digestion and measurements of soluble bioactive compounds across a membrane barrier. Some of these *in vitro* assays incorporate mammalian cell lines, such as the Caco-2 human intestinal epithelial cell line, and are commonly employed as alternatives to animal studies (9). The Caco-2 intestinal cell model is the most widely used and validated intestinal epithelial cell model, and although the cell line is colonic in origin, Caco-2 cells undergo spontaneous differentiation in cell culture to form a monolayer of polarized cells that display many of the functional and morphological properties of mature human enterocytes (5). The human HT29-MTX-E12 cell line is also widely used in mimicking human intestinal processes. The HT29-MTX-E12 cell lines are the human HT29 colorectal adenocarcinoma cells differentiated into mature goblet cells using methotrexate. Clones were then selected (E12) from these differentiated cells which possess the characteristics of human goblet cells. The characteristics of the cells include tight junction formation, development of confluent monolayers, and production of mucous layer (10).

*Terminalia ferdinandiana*, or Kakadu plum, is a native Australian fruit which is well known for its high levels of vitamin C [or ascorbic acid (AA)]. *T. ferdinandiana* also contains ellagic acid (EA) and calcium (Ca^+2^) and antinutrients such as oxalic acid (OA) (11). Increased uptake of OA has been reported to increase the development of kidney stones in susceptible people. Furthermore, oxalate is produced during mammalian metabolic processes, such as the breakdown of dehydroascorbic acid (i.e., oxidized AA), causing increased accumulation of OA (12). Soluble oxalates can bind with Ca^2+^ and form insoluble salts that limit bioavailability of Ca^2+^ and reduce free intracellular Ca^2+^ potentially leading to Ca^2+^ deficiency (12).

Very few is known about the fate of bioactive compounds from *T. ferdinandiana* during digestion. Subsequently, it is unknown whether EA, AA, and Ca^2+^, and antinutrients such as OA, are released from the *T. ferdinandiana* fruit matrix under physiological conditions *in vivo*, or whether digestion impacts stability of these compounds. When considering digestive stability of one component, it is important to consider not only the chemical structure of the component, but also the nature of its bond to the food matrix (13). Therefore, static and/or dynamic *in vitro* digestion models that mimic the human gastrointestinal digestion process are a common approach to determine matrix release (bioaccessibility) and stability of polyphenols, vitamins, minerals, nutrients, and nonnutrients in foods as an initial measure to predict potential bioavailability (13).

To the best of our knowledge, there are no previous studies evaluating the *in vitro* bioaccessibility and intestinal absorption of bioactive compounds such as EA, AA, and Ca^2+^, and antinutrients such as OA, from *T. ferdinandiana*. Therefore, to enhance existing knowledge of the bioaccessibility and absorption of EA, AA, OA, and Ca^2+^ from commonly consumed *T. ferdinandiana* foods, an *in vitro* digestion model (INFOGEST) and Caco-2-HT29-MTX-E12 coculture cell absorption model were applied.

## Materials and Methods

### Chemicals

Pepsin (porcine gastric mucosa) and bovine bile were obtained from Sigma-Aldrich (Castle Hill, NSW, Australia), pancreatin from porcine pancreas was obtained from MP Biochemicals (Irvine, CA, USA), sodium chloride, potassium chloride, sodium bicarbonate, and calcium chloride were obtained from Merck (Bayswater, VIC, Australia), and total bile acids (TBAs) assay kit (colormetric) was purchased from Sapphire Bioscience Pty Ltd. (Redfern, NSW, Australia). Nunc cell culture flasks and 96-well plates were purchased from Sigma-Aldrich (Castle Hill, NSW, Australia). Dulbecco's modified eagle medium (DMEM), Dulbecco's phosphate-buffered saline without calcium and magnesium (PBS), Hank's balanced salt solution (HBSS), penicillin and streptomycin, heat-inactivated fetal bovine serum (FBS), glutamax, trypsin-EDTA, nonessential amino acids (NEAA), and trypan blue exclusion dye were purchased from Invitrogen (Thermo Fisher Scientific Corporation, Carlsbad, CA, USA). F96 MicroWell™ Black polystyrene plates were purchased from Thermo Scientific (Waltham, MA, USA). CyQUANT^®^ NF assay reagent was purchased from Invitrogen (Molecular Probes, Thermo Fisher Scientific Corporation, Carlsbad, CA, USA). The Caco-2 cell line was purchased from the American Type Culture Collection (Manassas, VA, USA). The HT29-MTX-E12 cell line was purchased from Sigma-Aldrich (Castle Hill, NSW, Australia). Costar Transwell^®^ 24-well polystyrene plates were purchased from Corning Incorporated (Kennebunk, ME, USA).

### *T. ferdinandiana* Samples

Freeze-dried *T. ferdinandiana* powder (0.5 g) was mixed with 20 mL milli-Q water, vortexed, and used as the initial starting material for *in vitro* gastrointestinal digestion based on the INFOGEST model detailed in Section INFOGEST Gastrointestinal *in vitro* Digestion (14). Digestions were performed in duplicate with samples collected at various time points throughout the digestion process. Gastric digesta samples were collected at *t* = 0 (G0) and *t* = 60 (G60) min into the gastric phase, and intestinal digesta were collected at *t* = 30 (I30), *t* = 60 (I60), and *t* = 120 (I120) min into the intestinal digestion phase. The content of AA, EA, OA, and calcium in the freeze-dried *T. ferdinandiana* powder (starting material) was 21.1 g/100 g dry weight (DW), 2.8 /100 g DW, 1.4 g/100 g DW, and 295 mg/100 g DW, respectively (15, 16).

### INFOGEST Gastrointestinal *in vitro* Digestion

**Figure 1:**
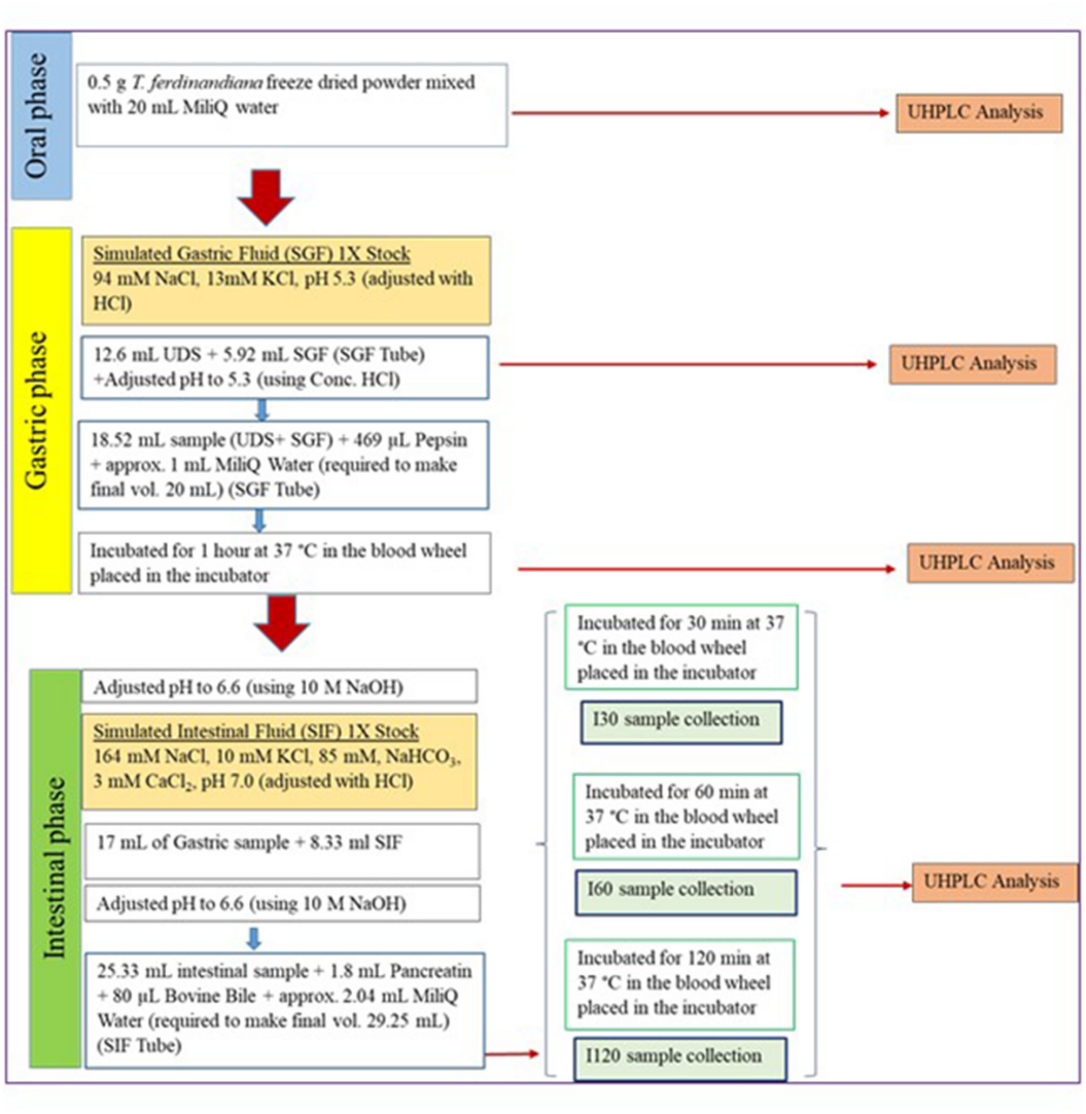
Schematic flow chart of the applied *in vitro* digestion protocol (14). SGF, simulated gastric fluid; UDS, undigested samples; SIF, simulated intestinal fluid.

A harmonized *in vitro* gastrointestinal digestion method developed in the COST action INFOGEST network was applied (14). Oral phase was not included in this experiment as *T. ferdinandiana* fruit is currently marketed as freeze-dried powder that would not undergo a substantial oral phase or may be incorporated into a nutraceutical product in pill, capsule, or beverage form. The digestive fluids (simulated salivary fluid (SSF), simulated gastric fluid (SGF), and simulated intestinal fluid (SIF)) were prepared according to the INFOGEST method (14). The activity of all enzymes was also measured according to the protocols described in the INFOGEST method (14) to ensure standardized application. Pepsin was used at a concentration of 268 units/ mL. Porcine pancreatin was used at 16 units/mL trypsin activity. Bovine bile equivalent to 1.38 mM was also added. Figure 1 provides an overview of the digestion protocols and conditions employed in the study. At the end of each digestion steps, samples were collected and snap-frozen (−80°C) to stop the digestion. Pefabloc at a concentration of 0.1 M was recommended to stop intestinal digestion; however, considering the cytotoxic nature of Pefabloc at a concentration ≥ 0.25 mM, Pefabloc was not applied to avoid future cell toxicity.

### Cell Culture

Caco-2 and HT29-MTX-E12 cells were grown in DMEM supplemented with 10% FBS (v/v), 1 X NEAA, 100 U/mL penicillin, 100 μg/mL streptomycin, and 2 mM glutamax in vented culture flasks at 37°C and 5% CO_2_. Cells were passaged every 2–3 days upon reaching 90% cell confluency and maintained within passage 10–25 for both cell types.

### Cytotoxicity of Digesta Samples

*In vitro* cell viability assays were employed to determine the dilution of digesta required to produce a noncytotoxic response throughout the *in vitro* absorption assays. Cell viability assays, cell plating, and media and buffer changes were performed on an epMotion^®^ 5075t liquid handling system (Eppendorf, Hamburg, Germany). Caco-2-HT29-MTX-E12 cocultures were prepared by mixing cells at a ratio of 9:1. Caco-2 cells (9,000) and HT29-MTX-E12 (1,000) cells were added in 100 μL media to each well in a Nunc™ F96 MicroWell™ Black polystyrene plate and incubated for 7 days at 37°C and 5% CO_2_. On the day of experiment, culture media was removed and replaced with 100 μL HBSS and incubated for 2 h. After 2 h, HBSS was removed and replaced with 50 μL intestinal digesta samples (I30, I60, and I120) diluted 2:1, 5:1, and 10:1 with HBSS, or HBSS for the control wells. Digesta samples were centrifuged with supernatants applied to the cells. The plates were incubated for 2 h at 37°C and 5% CO_2_. After 2 h, test compounds were removed and wells were washed with 100 μL HBSS. The HBSS was removed before 74 μL 1X CyQUANT^®^ NF dye binding solution was added to each well using a manual multichannel pipette. The plate was covered and incubated at 37°C for 60 mi. Fluorescence was measured with excitation at 485 nm and emission detection at 530 nm using a Spectramax M3 multimode microplate reader (Molecular Devices, San Jose, CA, USA).

### Caco-2-HT29-MTX-E12 Coculture

Caco-2 and HT-29-MTX-E12 cells were seeded on Costar Transwell^®^ plate inserts in a ratio of 9:1 and at a density of 1,09,000 cells/cm^2^ and 12,120 cells/cm^2^, respectively. Cells were cultured in growth media at 37°C and 5% CO_2_ for 21 days to facilitate cell differentiation and formation of an intact monolayer. During the 21-day differentiation period, growth media was removed and replaced with fresh media every 2–3 days. Cell differentiation of an intact monolayer was monitored by measuring the transepithelial electrical resistance (TEER) of the cell monolayer in an apical–basolateral direction. TEER was measured using the Millicell-ERS Voltohmmeter from Millipore (Burlington, MA, USA). TEER is a measurement of resistance and is a sensitive and convenient method to assess and the integrity of the cell monolayer. The values obtained are determined by cellular resistance (between apical and basolateral membrane) and the paracellular resistance (tight junctions). TEER values above 300 Ω.cm^2^ are indicative of differentiated Caco-2-HT29-MTX-E12 cells and an intact monolayer (17).

### Intestinal Absorption Assay

*In vitro* absorption was performed by following the procedure described by Osborne and colleagues (18). On the day of the intestinal absorption assay, TEER was measured and growth media was replaced with HBSS in the apical and basolateral chambers for 2 h to enhance uptake of the applied digesta. For the absorption study, intestinal digesta samples (I30, I60, and I120) were diluted 10:1 with HBSS. This dilution was selected based on the results from the cytotoxicity assay that showed no change to cell viability in response to digesta samples diluted 10:1. In the apical chamber, 200 μL diluted intestinal digesta (I30, I60, and I120) were applied for 120 min. HBSS (600 μL) was added in the basolateral chamber. After 2 h, all apical and basolateral samples were collected and stored at−80°C. HBSS was replaced with growth media in all chambers with TEER values measured again immediately after the assay. TEER was also recorded 24 and 48 h after the assay. Intestinal absorption studies were performed in duplicate. The rate of EA, AA, OA, and Ca^2+^ movement across the transwell^®^ membrane was determined by calculating the apparent permeability coefficient (*P*app) in cm/s as follows (19):


(1) $$\begin{array}{r} p_{app}=\frac{\Delta \text{Q}}{\Delta \text{t}}\times \frac{1}{A\times C_{0}} \end{array}$$

ΔQ/Δt is steady-state appearance rate of compound in receiver (basolateral) compartment (μmol/s).

A is the surface area of the filter (i.e., 0.33 cm^2^).

C_0_ is the initial concentration in the donor (apical) compartment (μM).

### Preparation and Analysis of Digesta Samples in UHPLC

#### Ascorbic Acid

*T. ferdinandiana* digesta and intestinal absorption samples were analyzed for AA. The samples were centrifuged and filtered through 0.22 μm syringe filters prior to analysis. An Acquity UHPLC system (Waters Corp., Milford, MA, USA), equipped with a Waters Acquity UHPLC photodiode array (PDA) detection system, was used to carry out the analysis. Empower™ software (Waters Corp., USA) was used to process and quantify peaks after recording the signals. A Waters Acquity HSS T3 analytical column (100 mm × 2.1 mm, 1.8 μm particle size) (Waters Corp., USA), with an isocratic mobile phase of 0.1% aqueous (v/v) formic acid at a flow rate of 250 μL/min, was used with an injection volume of 2 μL. The absorbance was measured at room temperature at 245 nm.

#### Ellagic Acid

*T. ferdinandiana* digesta and intestinal absorption samples were analyzed for EA. All samples were centrifuged and filtered through 0.22 μm PTFE membrane filters (Millipore, CA, USA) before injection into the chromatographic system. Samples were analyzed on a Waters Acquity UHPLC system (Waters Corp., Milford, MA, USA) equipped with PDA detector. A Waters BEH Shield RP18 Column 130 °A, 1.7 μm (100 mm X 2.1 mm) from Waters Corporation (Milford, Massachusetts, USA) was used. Column temperature was maintained at 40°C, the injection volume was 2 μL, PDA spectrum was scanned from 200 to 400 nm with a rate of 2.5 Hz, and detection was carried out at 254 nm. A combination of mobile phase A and phase B was used during 23 min of elution. Formic acid 0.1% was used for mobile phase A (Milli-Q water) and phase B (acetonitrile) at a flow rate of 0.4 mL/min. The gradient program started at 90% mobile phase A for 1 min, followed by a linear gradient to 85% for 4 min, to 65% A at 7 min, then 50% in the next 7 min, then 10% for 4 min, hold at this composition for 1 min, and then 90% at 20 min before reequilibration for 3 min.

#### Oxalic Acid

*T. ferdinandiana* digesta and intestinal absorption samples were cleaned up with a SPE step (20) for OA analysis. Mixed reversed phase-weak anion exchange sorbent Strata^®^ SAX (55 μm, 70 Å, 500 mg/3 mL) was purchased from Phenomenex (Torrance, CA, USA) and used in this study. The SPE process was maintained through an Analytichem VAC ELUT SPS 24 Port Vacuum Manifold from Agilent Varian (Santa Clara, CA, USA). The SPE columns were sequentially conditioned with 3 mL of methanol and 3 mL of milli-Q water. Digesta and intestinal absorption samples at a volume of 1 mL dripped through the cartridges by gravity (with a flow rate of about 1 mL/min). The cartridges were washed with 3 mL of water and 3 mL of methanol. The columns were dried under maximum vacuum for 5 min prior to elution. Water with 1% HCl/H_2_O with 5% NH_4_OH (2 mL) was used as eluent (20). All extractions were performed in triplicate. Afterward, samples were filtered through a 0.2 μm syringe filter and injected in the chromatograph. A Phenomenex Synergi 2.5 μm Hydro-RP 100 Å (100 mm X 3.00 mm) column and isocratic conditions were used to elute OA. The mobile phase consisted of 200 mM phosphate buffer (pH 1.5) with a flow rate 0.1 mL/ min. Run time was 10 min. PDA spectrum was scanned from 190 to 400 nm with a rate of 2.5 Hz, and detection was carried out at 210 nm. Column temperature was maintained at 25°C, and injection volume was 2 μL.

#### Calcium

Levels of Ca^2+^ were analyzed by ICP-OES (Agilent 700, Australia) after hot block digestion (A.I. Scientific, Australia). Briefly, 0.5–2.0 g samples were accurately weighed into digestion tubes before 4 mL of high-purity nitric acid (69% v/v, Seastar Chemicals, Canada) was added. The digested samples were made up to 20 mL with high-purity water (Aqua Cure, England). Appropriate reference materials were used for quality control.

### Statistical Analysis

Data are presented as the mean percentage of six replicates ± standard error of the mean (SEM) for each treatment. Cytotoxicity is expressed as the percentage of viable cells remaining after extract treatment compared to the HBSS control. The *in vitro* gastrointestinal digestion of *T. ferdinandiana* was carried out two times for each compound in triplicate with results expressed as the mean ± SEM. One-way analysis of variance (ANOVA) was performed to determine significant differences (*p* ≤ 0.05) between the concentration of bioactive compounds in nondigested and digested *T. ferdinandiana* samples. Significant differences between two groups were determined using nonparametric unpaired *t*-test. One-way ANOVA with Tukey's multiple comparison was used when comparing groups. A significance level of *p* ≤ 0.05 was used for all tests. All statistical tests were conducted using GraphPad Prism version 8 (GraphPad Software, San Diego, CA, USA).

## Results and Discussion

### *In vitro* Digestion and Bioaccessibility

**Figure 2:**
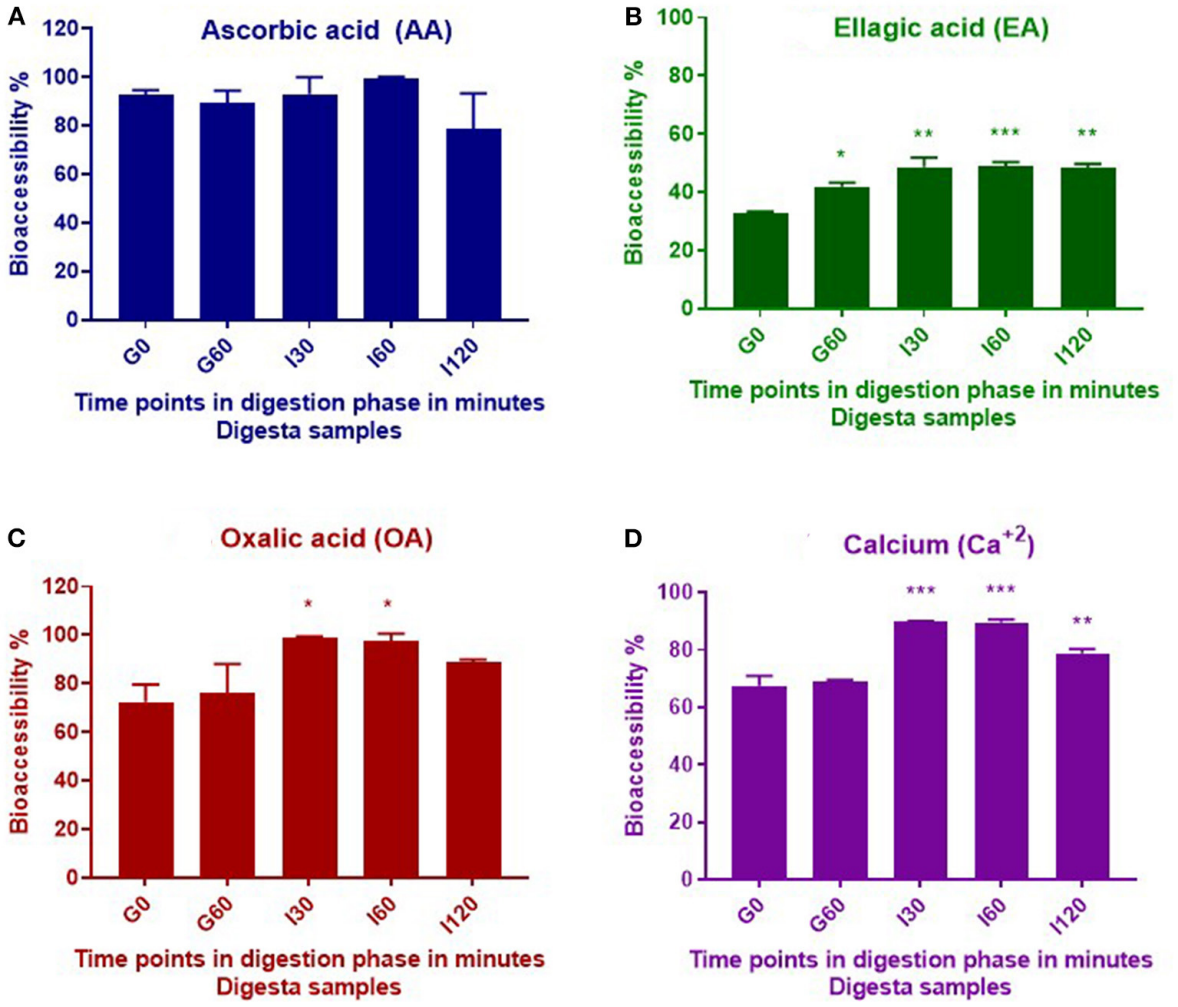
Bioaccessibility of AA **(A)**, EA **(B)**, OA **(C)**, and calcium **(D)** during *in vitro* digestion of *T. ferdinandiana* freeze-dried powder. Asterisks (*) indicate differences that are statistically significant between gastric *t* = 0 (G0) digesta and digesta from other stages of digestion (*p ≤ 0.01, **p ≤ 0.001, ***p ≤ 0.0001). G0, gastric 0 min; G60, gastric 60 min; I30, intestinal 30 min; I60, intestinal 60 min; and I120, intestinal 120 min.

Bioaccessibility was calculated by expressing compounds released during digestion as a percentage of the initial and total concentration of the compound in the plant material before digestion. Statistical significance was determined throughout gastrointestinal digestion by comparing bioaccessibility of the compounds during the gastric and intestinal digestion phases with bioaccessibility measured from the initial gastric *t* = 0 (G0) digesta sample. No significant differences were observed in bioaccessibility of AA throughout the *in vitro* digestion; however, OA and Ca^+2^ showed significant (*p* ≤ 0.05) increases in bioaccessibility throughout the intestinal phase, with respect to the G0 digestion phase, whereas EA significantly increased throughout all digestion phases (Figure 2).

#### Ascorbic Acid

Previous studies by our research group report the amount of AA in *T. ferdinandiana* fruits to be 21.1 /100 g DW (15). Results from this study indicate no significant changes (*p* > 0.05) in total AA content throughout *in vitro* digestion, suggesting that AA was not degraded during this process. AA is a water-soluble antioxidant and this property may account for the almost complete release of AA observed in this study from the solid to liquid phase. This is evident from the calculated bioaccessibility of 93% at the beginning of the gastric phase (G0) (Table 1). Approximately 90% of AA was then released from the solid phase into the liquid phase following digestion with pepsin for 60 min (G60). Digestion with pancreatin and bile in the small intestinal phase resulted in 93%, 100%, and 79% bioaccessibility after 30, 60, and 120 min, respectively (Figure 2A). Bioaccessibility of AA decreased toward the end of the intestinal phase; however, an unpaired *t*-test between AA bioaccessibility at I60 and I120 did not reveal any significant differences. Our present findings are in agreement with previous findings that reported minimal impact on AA stability during *in vitro* gastric conditions (at pH 2 or 3) (21). Rodríguez-Roque et al. (2) have also demonstrated that gastric digestion had few effect on AA stability by recovering 83% of this bioactive compound in a blended fruit juice containing orange, pineapple, and kiwi.

**Table 1:** Bioaccessibility of AA, EA, OA, and calcium (Ca^2+^) released from the *T. ferdinandiana* fruit matrix.

	**AA (%)**	**EA (%)**	**OA (%)**	**Ca^**2+**^ (%)**
Gastric *t* = 0	93 ± 2^a^	33 ± 1^b^	72 ± 7^c^	67 ± 4^c^
Gastric *t* = 60	90 ± 5^a^	42 ± 1^b^	85 ± 2^a^	69 ± 1^a^
Intestinal *t* = 30	93 ± 7^a^	49 ± 3^b^	99^a^ ± 0.0	90^a^ ± 0.0
Intestinal *t* = 60	100 ± 1^a^	49 ± 1^b^	98 ± 3^a^	90 ± 1^c^
Intestinal *t* = 120	79 ± 5^a^	48 ± 1^b^	89 ± 1^a^	79 ± 2^a^

*Data are expressed as the mean ± SD (n = 2). Bioaccessibility (%) = bioaccessible content/total content x 100. Statistical analysis consisted of comparing the bioaccessibility of AA, EA, OA, and calcium (Ca^2+^) of T. ferdinandiana digesta within each digestion phase using one-way ANOVA. Different letters within rows indicate significant differences (p ≤ 0.05) between the analyzed compounds within each digestion phase*.

#### Ellagic Acid

Polyphenols occur in foods mainly as esters, glycosides, and polymers that cannot be absorbed intact but require hydrolysis by digestive enzymes, or intestinal microbiota, to degrade or metabolize these compounds into a form that can be absorbed across the intestinal barrier. It is estimated that 48% of polyphenols are “digested” in the small intestine, 42% in the large intestine, and 10% remain undigested and intact within the food matrix (22).

In this study, significant increases (*p* ≤ 0.05) were observed in the bioaccessibility of EA across the different digestive phases compared to early gastric phase (Figure 2B). The content of total EA in *T. ferdinandiana* fruit (starting material) was 2.8 /100 g DW (16). Bioaccessibility of EA significantly increased from 33% at the beginning of the gastric phase to 48% at the end of the intestinal phase (I120). The increase in EA bioaccessibility may be due to the release of EA from ellagitannins and other EA derivatives produced during digestion. Studies involving other fruits, such as pomegranate and raspberries, suggest that during *in vitro* gastrointestinal digestion of blended fruit juices, EA is liberated from ellagitannins (2). It is also possible that bile salts and pancreatin present in the intestinal phase helped release EA from the food matrix by contributing to the breakdown of complex ellagitannins and other EA derivatives to produce free EA. Similarly, the potential role of bile salts and pancreatin has been highlighted previously in the release and bioaccessibility of carotenes from processed carrots (23).

#### Oxalic Acid

Figure 2 shows OA bioaccessibility from *T. ferdinandiana* fruit powder. Total OA content in *T. ferdinandiana* fruits was previously reported to be 1.4/100 g DW (15). Bioaccessibility of OA was estimated to be 72% at the beginning of the gastric digestion phase (G0) (Table 1) before significantly increasing to 98% in the intestinal phase (I30). At the end of the intestinal phase (I120), OA bioaccessibility decreased to 89% but was not significantly different to OA released in the early gastric phase (G60) (Figure 2C). An unpaired *t*-test was also performed between OA bioaccessibility at I60 and I120; however, no significant differences were observed.

#### Calcium

The Ca^2+^ content in *T. ferdinandiana* fruit was previously reported to be 295 mg/100 g DW (15). The release of Ca^2+^ at the beginning of the gastric phase was 67% and remained steady throughout the gastric phase as with no significant difference observed between G0 and G60 (Figure 2D). Bioaccessibility of Ca^2+^ significantly increased to 90% after 30 (I30) and 60 (I60) min in the intestinal phase before significantly decreasing to 79% at the end of the intestinal phase (I120) (Table 1) (*p* ≤ 0.05). The decrease in the bioaccessibility of Ca^2+^ at the end of the intestinal phase can be correlated with the fact that calcium has a tendency to bind with fatty acids in the lumen forming insoluble soaps (24, 25). In the *in vitro* settings of this study, Ca^2+^ might bind with the bile components or bioactive compounds released from the matrix of *T. ferdinandiana* fruits and formed insoluble complexes and reduced the availability of free Ca^2+^.

The recommended dietary intake (RDI) of Ca^2+^ for adults is 840 mg/day in Australia (26). The Ca^2+^ content is high in *T. ferdinandiana* fruits (295 mg/100 g DW) (15) compared to some other Australian native fruits, such as lemon aspen (133.3 mg/100 g DW), Davidson's plum (217.3 mg/100 g DW), and Quandong (133.3 mg/100 g DW) (27), qualifying *T. ferdinandiana* as a good source of Ca^2+^. Bioavailability of Ca^2+^ from matrix-bound Ca^2+^ salts is reportedly influenced by gastric acid secretion and simultaneous ingestion of other foods (28). Cilla et al. (5) have suggested that mineral bioaccessibility can be enhanced by applying thermal treatments to foods to soften the food matrix and increase the release of protein bound minerals. Part of the *T. ferdinandiana* fruits pureeing process involves steaming and may have loosened the food matrix in this study helping to release Ca^2+^ throughout the gastric and intestinal phases of *in vitro* digestion.

### *In vitro* Cytotoxicity of Digesta Samples

**Figure 3:**
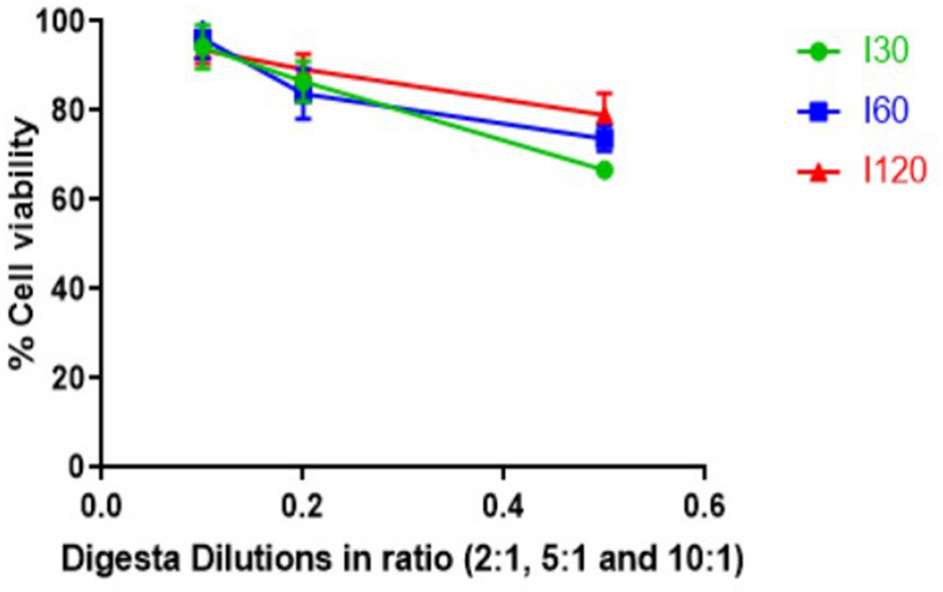
Effect of digesta prepared from *T. ferdinandiana* on Caco-2-HT29-MTX-E12 coculture cell viability *in vitro* using CyQUANT^®^ NF cell proliferation assay. Results are expressed as mean ± SEM of two independent experiments with 4 replicates in each (*n* = 8). I30, intestinal 30 min; I60, intestinal 60 min; and I120, intestinal 120 min.

Cytotoxicity in response to digesta samples was assessed *in vitro* with the CyQUANT^®^ NF cell viability assay using Caco-2-HT29-MTX-E12 cocultures. Figure 3 represents the impact of different dilutions (2:1, 5:1, and 10:1) of intestinal digesta samples (I30, I60, and I120) on the viability of Caco-2-HT29-MTX-E12 cocultures. Average cell viability in response to the digesta ranged from 67 to 79% for the 2:1, 84 to 89% for 5:1, and 94 to 96% for 10:1 dilution with no significant impact on cell viability observed in response to any of the diluted digesta. However, cell viability decreased when the digesta samples were more concentrated (Figure 3) with the 2:1 dilution having a greater impact on cell viability compared to 5:1 and 10:1 dilution. These cell viability results informed the selection of the dilution for the intestinal absorption study. Overall, the 10:1 dilution was selected for all digesta samples as cell viability was close to 100% in response to all 10:1 dilution.

### Transepithelial Electrical Resistance Measurement

Transepithelial electrical resistance is reflective of cell barrier integrity; therefore, TEER values were measured before and after the intestinal absorption assay, and again posttreatment at *t* = 24 and 48 h. All TEER values remained close to or above 300 Ω.cm^2^, indicating Caco-2-HT29-MTX-E12 cell viability and an intact monolayer. Maintaining a TEER value of ≥300 Ω.cm^2^ is an indication of cell monolayer integrity and ensures formation of a confluent cell monolayer with well-established tight junctions that allow the passage of the compounds by the paracellular route (17). Lower values indicate that tight junctions between cells have not developed enough or are damaged such that small molecules could pass *via* a paracellular route to the basolateral chamber (29).

For all intestinal absorption studies, TEER values ranged from 309 to 413 Ω.cm^2^ before application of digesta, and between 273 and 370 Ω.cm^2^ after treatment. Overall, the average change in TEER before and after the intestinal absorption assays ranged from 1 to 5%. Constant TEER values suggest that application of the diluted digesta did not disrupt the monolayer that is associated with intestinal absorption *via* paracellular routes (30). A small number of wells showed reduced TEER values immediately after the treatment and may have been due to lack of serum in the coculture model. It has been reported that cells grown on transwell membranes in a medium without serum display a loosening, or even loss of tight junction (31). Based on the results from the cytotoxicity assay, it is possible that the decline in initial TEER values in this study was not due to cell death but due to a change in Caco-2-HT29-MTX-E12 permeability. TEER measurements 24 h postdigesta application showed increased values where decreases in TEERs were observed immediately after application. Consecutive measurements 48 h postdigesta application revealed steady TEER values that were similar to initial measurements further indicating that the diluted digesta samples were not toxic to the cells (31).

### Caco-2-HT29-MTX-E12 Intestinal Absorption Model

**Figure 4:**
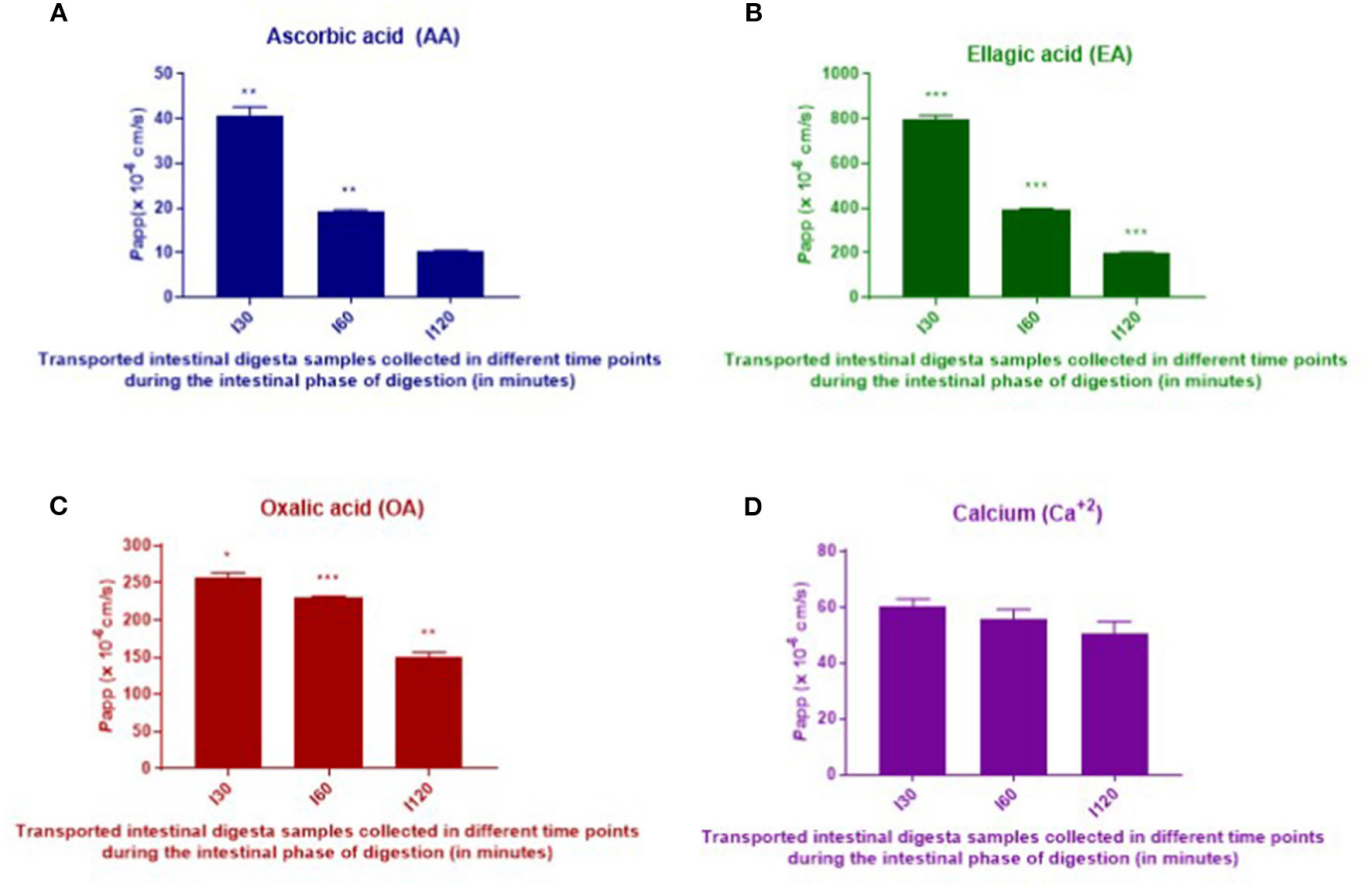
*In vitro* intestinal absorption of AA **(A)**, EA **(B)**, OA **(C)**, and calcium **(D)** from digested *T. ferdinandiana* powder across Caco-2-HT29-MTX-E12 cell barriers. Caco-2-HT29-MTX-E12 cocultures grown on transwells were incubated with I30, I60, and I120 intestinal digesta at 10:1 dilution for 2 h. Results are expressed as the mean ± SEM of 2 determinations (*n* = 2). One-way ANOVA followed by Tukey's multiple comparison tests was performed to compare apparent permeability (*P*app) for each compound from I30, I60, and I120. Asterisks (*) indicate differences that are statistically significant among the applied digesta (I30, I60, and I120) samples within each compound (*p ≤ 0.01, **p ≤ 0.001, ***p ≤ 0.0001). I30, intestinal 30 min; I60, intestinal 60 min; and I120, intestinal 120 min.

Intestinal absorption of AA, EA, OA, and Ca^2+^ from digested *T. ferdinandiana* powder was measured using Caco-2-HT29-MTX-E12 coculture cell monolayers. Intestinal digesta samples that were collected at different time points (I30, I60, and I120) throughout the *in vitro* digestion were applied to the coculture transwell model for 120 min before apical and basolateral samples were collected. The amount of AA, EA, OA, and Ca^2+^ was measured in the digesta initially applied to the apical chamber of the transwell model, and in samples collected from the basolateral chamber. Apparent permeability (*P*app) was calculated with statistical significance observed (*p* ≤ 0.05) in *P*app values obtained from the applied I30, I60, and I120 digesta samples (Figure 4 and Table 2). Overall, *P*app of absorbed AA, EA, and OA was significantly higher from I30 digesta compared to I60 and I120 digesta. However, absorption of Ca^2+^ did not significantly change when comparing any digesta.

**Table 2:** Apparent permeability (*P*app) of AA, EA, OA, and calcium (Ca^2+^) from *T. ferdinandiana* digesta.

**Samples**	**AA**	**EA**	**OA**	**Ca^**2+**^**
	***P*app (x 10^**−6**^ cm/s)**	***P*app (x 10^**−6**^ cm/s)**	***P*app (x 10^**−6**^ cm/s)**	***P*app (x 10^**−6**^ cm/s)**
I30	41 ± 5^a^	796 ± 7^a^	258 ± 5^a^	60 ± 6^a^
I60	19 ± 0.4^b^	394 ± 4^b^	231 ± 1^b^	56 ± 7^a^
I120	10 ± 0.1^b^	201 ± 2^c^	150 ± 7^c^	50 ± 9^a^

*Data are expressed as the mean ± SD (n = 2). Statistical analysis consisted of comparison of I30, I60, and I120 digesta samples within each compound using one-way ANOVA with Tukey's multiple comparison. Different letters within columns indicate significant differences (p ≤ 0.05)*.

*In vitro* digestion and Caco-2-HT29-MTX-E12 cell monolayer models were utilized in this study to determine *in vitro* absorption as an indicator for bioavailability, of selected bioactive compounds in *T. ferdinandiana* fruit powder. In general, bioavailability of bioactive compounds depends on digestive stability and efficiency of the transepithelial passage. Generally, *P*app values <1 x 10^−7^ and >1 x 10^−6^ cm/s in an apical (AP) to basolateral (BL) direction (AP to BL) are considered low and high permeation, respectively (32). According to the traditional Caco-2 cell assay used to investigate intestinal absorption of drugs, *P*app values > 3 x 10^−6^ cm/s suggest highly permeable compounds and represent an intestinal absorbed fraction in humans of more than 60% whereas *P*app values <3 x 10^−6^ cm/s are characteristic of low permeability compounds representing an intestinal absorbed fraction in humans of <60% (17). Another study reports that the intestinal absorption of xenobiotics is considered negligible if the *P*app is <0.1 X 10^−6^ cm/s and essentially complete if the transepithelial *P*app is >5.0 X 10^−6^ cm/s (33). The *P*app values obtained for EA were higher than those calculated for AA, OA, and Ca^2+^. The *P*app values of EA measured in this study are higher than the *P*app values of EA (0.347 ± 0.018 /10^−6^ cm/s) reported previously (34). An interesting finding from the study presented here is that *P*app values were higher in response to I30 samples compared to I60 and I120 samples.

From the *P*app observed in this study, it is apparent that EA, AA, OA, and Ca^2+^ demonstrated significant intestinal absorption. However, *in vitro* results cannot replace *in vivo* studies, and there are several factors that can affect intestinal absorption of bioactive compounds *in vivo*. Compound loss during gastric digestion, pH changes, solubility, interactions with other compounds and enzymes, food matrix, removal by blood stream, metabolism, and microbial breakdown of complex compounds can all affect bioavailability of the compound (32).

With respect to intestinal absorption of EA *in vitro, P*app of 1 mg/mL EA across a Caco-2 cell barrier (at a density of 1.2 x 10^5^ cells/insert in a 12-well transwell plate) for 4 h in an absorptive direction (apical–basolateral) was previously reported to be 0.347 x 10^−6^ cm/s (34). The *P*app values obtained in this study are higher than the previous report. In the previous report, Caco-2 cell lines were used, and in this study, a coculture of Caco-2-HT29-MTX-E12 was used. The use of a coculture of Caco-2-HT29-MTX-E12 could accurately imitate the small intestine because the tight junctions of HT-29 cells are not as tight as the Caco-2 cells (35). Moreover, the generation of mucus and the TEER value in a coculture of Caco-2-HT29-MTX-E12 are more similar to that of human small intestine (35). Additionally, for compounds undergoing passive intestinal absorption, permeabilities were generally higher in cocultures than in Caco-2 monolayers (36). Lan et al. (37) reported that absorption of EA by HepG2 cells was detected just 15 min after application with peak absorption measured after 4 h. Bioactive compounds encounter several challenges throughout ingestion, digestion, and absorption across the intestinal barrier. There are numerous reports on the *in vitro* absorption behavior of different compounds with some reports based on Caco-2 monolayers, and some on a coculture system involving Caco-2 and HT29-MTX-E12 cells.

Based on the previous findings, the freeze-dried *T. ferdinandiana* powder (1 g) used in this study was predicted to contain 210 mg of AA. Based on bioaccessibility being 100% for AA at I60, it can be inferred that a recommended serve of *T. ferdinandiana* freeze-dried powder (i.e., 1 g) could provide an adequate amount of bioaccessible AA compared to the minimum RDI of 45 mg per day, but would still be within the upper intake level of 2,000 mg/day (26). However, the low apparent permeability of AA measured in this study could suggest reduced levels of AA for distribution to tissues. In this study, bioaccessibility of EA only reaches a maximum of 49% in the I30 and I60 phases; however, apparent permeability was higher compared to the other phytochemicals tested.

## Conclusion

*T. ferdinandiana* is a nutritious fruit with potential health benefits that could alleviate chronic human diseases. However, no previous studies have investigated bioaccessibility and intestinal absorption of important bioactive compounds, such as EA, AA, OA, and Ca^2+^, present in *T. ferdinandiana*. Findings from this study showed that each compound exhibited different bioaccessibilities and intestinal absorption *in vitro*. The intestinal absorption assay revealed that EA had the highest permeability *in vitro*. This study also demonstrated that even though bioaccessibility of AA was high, *in vitro* permeability was low. *In vitro* experiments do not always reflect the complex *in vivo* environment; however, *in vitro* models can be valuable screening tools for informing *in vivo* trials. The challenges that impact bioaccessibility and intestinal absorption of different *T. ferdinandiana* compounds, such as solubility, permeability, and cell accumulation, need to be addressed when formulating food products and additives using *T. ferdinandiana* fruit. The findings from this study have provided novel and important data that could be used to predict *in vivo* bioavailability of EA, AA, OA, and Ca^2+^ from *T. ferdinandiana*. This will also assist in the development of healthy and functional food products based on *T. ferdinandiana* fruit.

## Data Availability Statement

The original contributions presented in the study are included in the article/supplementary material, further inquiries can be directed to the corresponding author/s.

## Author Contributions

SA, MN, UT, MF, YS, and SO conceived and designed the study. SA performed the experiments, analyzed data, and wrote the manuscript. RA helped with the experiments. UT helped with the calcium analysis. MN, MF, YS, and SO critically revised and edited the manuscript. All authors approved the final manuscript.

## Funding

This project was funded by AgriFutures Australia Grant 201430161, and by the CSIRO Active Integrated Matter Future Science Platform. SA was supported by an Australian Government Research Training Program Scholarship and the University of Queensland.

## Conflict of Interest

The authors declare that the research was conducted in the absence of any commercial or financial relationships that could be construed as a potential conflict of interest.

## Publisher's Note

All claims expressed in this article are solely those of the authors and do not necessarily represent those of their affiliated organizations, or those of the publisher, the editors and the reviewers. Any product that may be evaluated in this article, or claim that may be made by its manufacturer, is not guaranteed or endorsed by the publisher.
